# Simulation and estimation of future ecological risk on the Qinghai-Tibet Plateau

**DOI:** 10.1038/s41598-021-96958-5

**Published:** 2021-09-02

**Authors:** Shengzhen Wang, Fenggui Liu, Qiang Zhou, Qiong Chen, Fei Liu

**Affiliations:** 1 College of Geographical Sciences, Qinghai Normal University, Xining, 810000 Qinghai China; 2 Academy of Plateau Science and Sustainability, Xining, 810000 Qinghai China

**Keywords:** Climate sciences, Environmental sciences

## Abstract

Over the past 50 years, temperatures on the Qinghai-Tibet Plateau (QTP) have risen roughly twice as fast as the global average, making it the most unpredictable region of environmental change due to global warming. In this paper, an Environmental Area Index model was developed using data from the Coupled Model Intercomparison Project to assess the ecological risk faced by QTP ecosystems under the influence of climate factors. The results show that ecological risk gradually decreases from northwest to the southeast, and there are different trends in ecological risk for each class in areas with different elevation gradients. As elevation increased, the proportion of potential risk areas gradually decreased, and the proportion of high- and higher-risk areas gradually increased. We predict that in the period 2021–2100, the overall ecological risk change trend on the QTP will not be obvious, but there will be a more obvious change on the vertical gradient. In general, under the existing global climate change scenario, the ecological risk faced by the QTP show a decreasing trend under the influence of climate factors, and the decrease in ecological risks is much higher at higher elevations than at lower elevations.

## Introduction

Global climate change is characterized by rising temperatures and is already seriously affecting natural ecosystems in many parts of the world^1–4^. The Qinghai-Tibet Plateau (QTP) is the highest tectonic unit in the world and an important ecological barrier for China, as well as Asia as a whole^5–8^. However, its high altitude, low temperature, strong radiation, numerous rivers and lakes, extensive glacial permafrost, and rich biodiversity determine its extremely high environmental vulnerability^9–18^. The Fifth Assessment Report of the Intergovernmental Panel on Climate Change (IPCC; 2014) revealed that the average temperature on the QTP exhibits a clear upward trend, with the rate of warming in that area over the past 50 years exceeding the global average rate by a factor of two^19–23^. With global warming, glaciers and permafrost have changed significantly in certain sensitive areas, accelerating the degradation of ecosystems^2,3,24,25^. Climate change-driven ecosystem changes on the QTP are primarily manifested in the reduction and shrinkage of the alpine meadow vegetation^26,27^, the early return of plateau vegetation^12,19,28–30^, increased ecosystem productivity^31–34^, and the tree line lift^35^, which have an important impact on the energy and carbon balance across both the QTP as well as the globe^36,37^; These climate-change-driven changes on the QTP further affect the global climate, energy cycles, and water cycles^38^. Thus, the QTP ecosystem's response and feedback to future climate change is not just related to the ecological security of the QTP itself but is also of great importance to the ecological environment and climate of adjacent regions^1^.

However, the distribution of ecological risks and their changes on the QTP due to climate change as the main drivers are currently unclear^39^. Keeping in mind the aforementioned objectives, this paper uses temperature, precipitation, vegetation and other data from different SSP scenarios (SSP126 scenario, SSP245 scenario, SSP370 scenario, SSP585 scenario) of the Sixth Assessment Model of the IPCC (Coupled Model Intercomparison Projects)^40,41^ to calculate the climate quality index (CQI), vegetation quality index (VQI) and soil quality index (SQI). These indices were used to construct the Environmental Area Index (EAI) model for assessing the risk of the QTP based on the ESAI risk assessment model^42–44^. We then used it to evaluate the future ecological risk of the QTP and explored possible changes in the next hundred years with a view to providing a reference for human interventions, ecological barrier protection strategies, and conservation projects.

## Materials and methods

### Data source

The evaluation index data are primarily based on model simulation, remote sensing, soil, and land use data. The names of the datasets corresponding to each index as well as their download sources are shown in Table 1. Future climate and other indicators are selected form the BCC-CSM2-MR, CNRM-CM6-1, CNRM-ESM2-1, CanESM5, GFDL-ESM4, IPSL-CM6A-LR, MIROC-ES2L, MIROC6 and MRI-ESM2-09 models from the CMIP6^41^.

**Table 1:** Data sources of ecological risk assessment indicators for the QTP.

	Name	Data
Scenario data	SSP126, SSP245, SSP370, SSP585 scenario data	CMIP6 pattern scenario dataset (https://esgf.llnl.gov/)
Climate factors	Temperature	China Meteorological Background Data Set (http://www.resdc.cn)
	Precipitation	China Meteorological Background Data Set (http://www.resdc.cn)
	Accumulated temperature	China Meteorological Background Data Set (http://www.resdc.cn)
	Drought index	Global Drought Map—10 Arcs (http://www.fao.org/geonetwork/)
	Photosynthetically active radiation	China-ASEAN 5 km resolution photosyntheticall effective radiation data set (http://www.geodoi.ac.cn/)
Soil factors	Soil texture	Data on the spatial distribution of soil texture in China
	Soil erosion (wind, hydro, freeze–thaw)	Spatial distribution of soil erosion in China (http://www.resdc.cn)
	Soil organic matter	China 1:1 million soil organic matter content
	Soil depth	China Soil Data Set of the World Soil Database (HWSD) (v1.1) (http://westdc.westgis.ac.cn/zh-hans/)
Vegetation factors	Vegetation coverage	Global Land Surface Feature Ginseng (GLASS)—Vegetation Cover
	Richness	China 1 km Bioffenge Index Data Set (http://www.geodoi.ac.cn/)
	NPP	Based on the light energy utilization model GLM_PEM obtained from the calculation
	The type of vegetation	China 1:1 million vegetation type datasets (http://www.resdc.cn)
Terrain factor	Elevation	Digital elevation data dem extraction (http://www.gscloud.cn/)
	Slope	Digital elevation data dem extraction (http://www.gscloud.cn/)

To facilitate the calculation and analysis, the data format and accuracy were standardized using ArcGIS software, which converted vector data to raster data and resampled all raster data to a cell size of 500 m.

### Methods

The Environmentally Sensitive Areas Act (ESAs) Act^42,43,45^ is a model that adapts to a variety of environmental quality factors, such as terrain, climate, vegetation, soil, population, and economy; it is mainly used for qualitative evaluations of ecologically sensitive areas in the field of ecological protection. Based on the ESA method and model as well as the special geographical and environmental characteristics of the QTP, this paper constructs a quantitative evaluation model based on ecological factors that summarize the natural factors that constituting the ecological risk of climate change in climate index (CQI), soil index (SQI) and vegetation index (VQI). Subsequently, an ecological risk assessment model (EAI) under the influence of natural factors on the QTP is established to estimate the ecological risk of future climate change on the QTP.

During this study, the referenced time periods are based on data from the IPCC: the base period is 1961–2015, the forecast period is divided into four sections, namely, 2021–2040, 2041–2060, 2061–2080 and 2081–2100, and discussed at an average of 20 years.

#### Climate Quality Index (CQI)

Indicators for calculating the CQI were selected based on that in the original ESA model^42^. The selected factors included the average annual precipitation (a key variable affecting vegetation growth) (mm year^−1^), the drought index (mm mm^−1^) which include the long-term average precipitation and potential evapotranspiration factors. The special characteristics of the QTP are fully considered to form a calculation method that specifically caters to the CQI applicable to the QTP.

Another important factor influencing plant growth and biomass distribution is the ability of green plants to synthesize organic compounds based on photosynthetically active radiation (PAR). In China, QTP possesses the most abundant opportunities for sunlight. The loss of solar radiation before it hits the ground at the QTP is relatively lower due to its high altitude and many sunny days. PAR is, therefore, an important factor for evaluating climate quality on the QTP.

Accumulated temperature is another indispensable factor when evaluating local climate quality as well as characterizing a region's thermal conditions. It is thus also one of the indicators for measuring the thermal conditions required for crop growth and development; an accumulated temperature ≥ 10 °C reflects the amount of heat required for crop growth. Slope is another factor that has a major effect on climate and as well as water and heat due to the unique geographical location and topographical characteristics of the QTP. Slope, therefore, is used as an auxiliary factor among climate components in the study.

Therefore, based on the aforementioned indices, the climate quality estimation model for the QTP is as follows:

1 $$ CQI = \left( {\Pr *T*Di*Al*Par*At} \right)^{\frac{1}{6}} $$

where *Pr* is the average annual precipitation, *T* is the annual average temperature, *Di* is the drought index, *Al* is altitude, *Par* is photosynthetically active radiation, and *At* is the accumulated temperature greater than 10 °C in the region. The climate quality indicators were then scored with reference to the study of reference^45^ based on the results calculated. The results are shown in Table 2.

**Table 2:** The scores of various indexes in the climate quality of the QTP.

	Classes	Scores		Classes	Scores
**Climate quality**					
Precipitation	< 100 mm	1.7	Altitude	> 5000 m	2.0
	100–200 mm	3.4		4400–5000 m	4.0
	200–400 mm	5.1		3600–4400 m	6.0
	400–600 mm	6.8		2300–3600 m	8.0
	600–800 mm	8.5		< 2300 m	10.0
	> 800 mm	10.0			
Temperature	< − 5℃	2.0	Photosynthetically active radiation	< 3300 MJ/m^2^	2.0
	− 5–− 3℃	4.0		3300–3500 MJ/m^2^	4.0
	− 3–0℃	6.0		3500–3700 MJ/m^2^	6.0
	0–10℃	8.0		3700–3800 MJ/m^2^	8.0
	> 10℃	10.0		> 3800 MJ/m^2^	10.0
Drought index	< 0.05 (Super drought)	2.0	Accumulated temperature (10 times)	< 500	1.25
	0.05–0.2 (Drought)	4.0		500–1000	2.50
	0.2–0.5 (Semiarid)	6.0		1000–2000	3.75
	0.5–0.65 (Dry and semi humid)	8.0		2000–3000	5.00
	> 0.65 (Moist)	10.0		3000–4000	6.25
				4000–5000	7.50
				5000–6000	8.75
				> 6000	10.00

**Figure 1:**
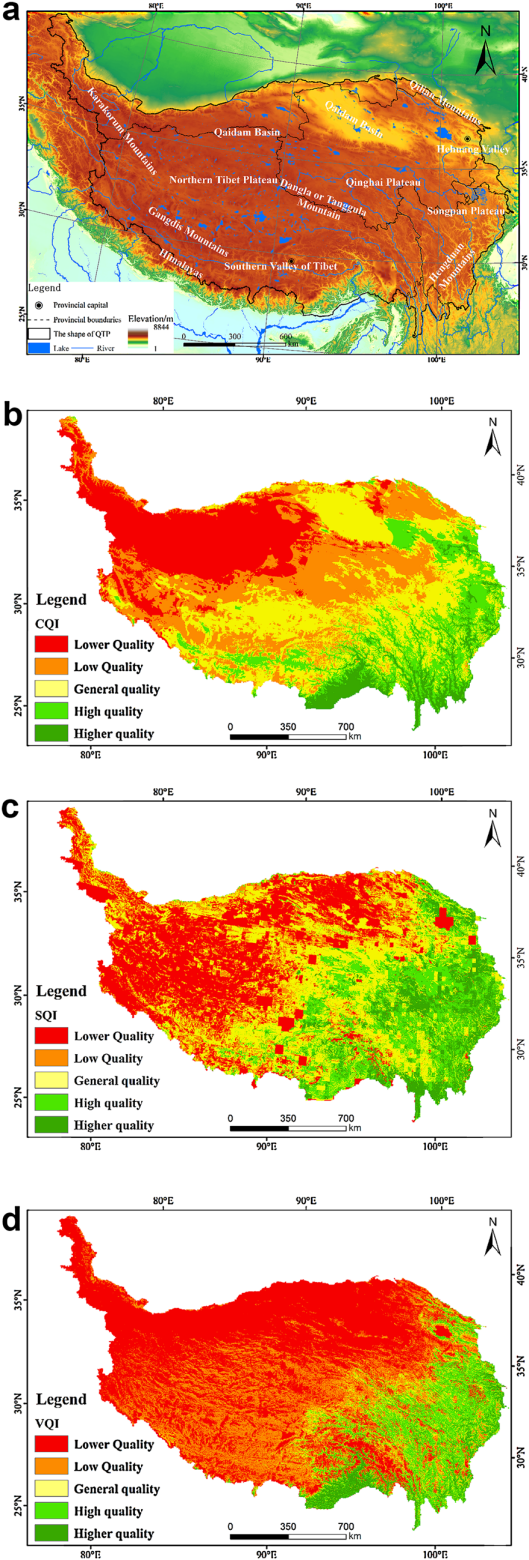
(**a**) Location of DEM over the QTP. The map was generated by ArcGIS sofware (Version 10.5 for Desktop). (**b**) The climate quality of the QTP. The map was generated by ArcGIS sofware (Version 10.5 for Desktop) for Desktop). (**c**) The soil quality of QTP. The map was generated by ArcGIS sofware (Version 10.5 for Desktop). (**d**) The vegetation quality of the QTP. The map was generated by ArcGIS sofware (Version 10.5 for Desktop).

The results reveal that the climate quality of the QTP (Fig. 1a, b) shows a gradually increasing trend from the northwest to the southeast, similar to the results of many other studies^46–49^. This climate quality is graded according to natural breaks, and the results (Table 3) reveal that on the QTP, the largest proportion of GCQ (general climate quality) area is 30.58%. The areas of GCQ are mainly located in the Qaidam Basin of the southern part of the southern Qinghai Plateau in the southern region of the Northern Tibetan plateau as well as the Gangdis Mountains. The major climates in these areas are temperate desert and temperate grassland, with a few areas possessing a sub-cold grassland climate. The area of the LeCQ (lower climate quality) climate zone accounts for 23.29% of the total QTP area and was mainly distributed in the northern Kunlun mountains of the northern Tibetan plateau, making it slightly smaller than that of the GCQ. The main climate types in these areas are boreal steppe and boreal desert. The proportion of the LCQ (low climate quality) area is 17.14% of the plateau; it is mainly located in the Qilian mountains, in the north-central part of the Qinghai Plateau, and in the central part of the northern Tibet Plateau. The main climate type of this area is sub-cold steppe. On the other hand, the proportion of the HCQ (high climate quality) area is 13.41%; it is mainly located in the Hehuang valley, Songpan Plateau, and the southern valley of Tibet. The proportion of the HeCQ (higher climate quality) area is 5.48%; it is mainly located in the Hengduan mountains and the southern valley of Tibet. The main climate types here are temperate deciduous broadleaf forest, temperate forest-steppe, and, in certain areas, northern subtropical monsoonal deciduous broadleaf-evergreen forest, central subtropical monsoonal evergreen broadleaf forest, and southern subtropical monsoonal rainforest.

**Table 3:** The climate quality and proportion of the QTP.

Climate quality		Percentage (percent)
Lower climate quality (LeCQ)	< 2.89	23.39
Low climate quality (LCQ)	2.89–3.82	17.14
General climate quality (GCQ)	3.82–4.85	30.58
High climate quality (HCQ)	4.85–6.21	13.41
Higher climate quality (HeCQ)	> 6.21	5.48

#### Soil Quality Index (SQI)

Soil is the material basis for human survival and an indispensable player in both the ecosystem and the carbon cycle^50,51^. It is an important natural resource that maintains the productivity of plants and animals, improves the water and air quality, and guarantees a healthy life for humans. Soil quality refers to the ability of a particular type of soil to function within the boundaries of either a natural or managed ecosystem. It is directly related to the social economy’s sustainable development as well as human health. The soil quality’s impact on the ecosystem is mainly determined by the water storage and retention capacity of the soil, its organic matter content, and the prevention of erosion.

Soil texture comprises soil particles of different sizes that make up the different pores in the soil. Soil containing more sand grains has larger pores; precipitation can easily infiltrate it and the internal drainage is fast. Soil with a higher number of clay particles has small pores, due to which its water content and nutrients are not easily lost^54^. Under the influence of the special climate, soil in most areas of the QTP is characterized by a thin layer (20–60 cm). The organic matter present in this soil has nutrient-rich characteristics. Therefore, when calculating the soil quality of the QTP, soil texture must be considered.

In addition to this, the slope is the driving force behind the horizontal flow of soil water and nutrients and has an important impact on the soil system. Rainfall infiltration conditions are poor in areas with relatively large slopes. However, a large slope will create good drainage conditions, which is conducive to runoff. In areas with relatively larger slopes, vegetation is sparser and has a low cover. The ability of these areas to absorb and retain soil moisture is weaker, resulting in the water movement down the slope. Conversely, the lateral movement of soil moisture is weaker, leading to long-term accumulation on the lower slopes and thereby affecting the process and storage of nitrogen accumulation in the area, which, in turn, impacts soil quality.

Based on the aforementioned reasons and the factors originally selected by the EAS model to calculate soil quality, the factors of soil depth, texture, moisture, and parent material were finally selected for analysis. Considering the geological peculiarities of the QTP, the soil erosion intensity was increased and the parent material was substituted using soil organic matter.

A model for calculating soil quality on the QTP was established as follows:

2 $$ SQI = (Sd*St*Sei*Som*Sm)^{\frac{1}{5}} $$

where *Sd* is the soil depth, *St* is the soil texture, *Sei* is the soil erosion intensity, *Som* is the soil organic matter, and *Sm* is the soil moisture. To determine the relationship between other factors and soil quality, deeper soil depths, which correspond to higher soil quality, were employed. All the indicators were scored according to various soil quality indicators provided by Salvati’s study (2013). The results are shown in Table 4.

**Table 4:** The scores of various indexes of soil quality in the QTP.

	Classes	Scores		Classes	Scores
**Soil quality**					
Soil depth	0	2.5	Soil texture	< 0.25	2.5
	10	5.0		0.25–0.5	5.0
	30	7.5		0.5–0.75	7.5
	100	10.0		> 0.75	10.0
Soil moisture (m^3^/m^3^)	< 0.06	2	Soil erosion	Severe	1.7
	0.06–0.077	4		Extreme slightly	3.4
	0.077–0.098	6		Strength	5.1
	0.098–0.122	8		Moderate	6.8
	> 0.122	10		Mild	8.5
				Slightly	10.0
Soil organic matter	< 0.8	2.0			
	0.8–2.7	4.0			
	2.7–5	6.0			
	5–9.2	8.0			
	> 9.2	10.0			

To classify the results of the measurement, the natural breaks method was used. The results showed that 67.32% of the soils on the QTP belonged to LSQ (low soil quality) and GSQ (general soil quality), while only 9.82% belonged to HSQ (high soil quality) (Table 5).

**Table 5:** The soil quality and its proportion in the QTP (percent).

Soil quality		Percentage (percent)
Lower soil quality (LeSQ)	< 3.88	35.09
Low soil quality (LSQ)	3.88–5.14	5.48
General soil quality (GSQ)	5.14–6.36	34.23
High soil quality (HSQ)	6.36–7.41	15.37
Higher soil Quality (HeSQ)	> 7.41	9.82

The area of LSQ is mainly located between the Kunlun Mountains and the Himalayas, including the northern Tibet Plateau and the Ali Plateau, where the main soil type is cold desert soil and mountain soil. The area of HSQ accounts for 15.37% and is mainly distributed in the southeastern Qilian mountains, Hehuang valley, a majority of the Songpan Plateau, the southern valley of Tibet, and a majority of the Hengduan mountains. The main soil types are mountain grassland, mountain meadow, and cultivated soil. The HeSQ (higher soil quality) area is mainly located in the southern Hengduan mountains, the southern Qinghai Plateau, and the southern Songpan Plateau, where the main soil type is forest (Fig. 1a,c). Some obvious red patches can be seen in the soil quality distribution map since the lakes was not excluded from the calculation.

#### Vegetation Quality Index (VQI)

Vegetation, as an important component of terrestrial ecosystems, exhibits the most prominent and significant response to global climate change^52,53^. Its ecological functions include rainfall interception as well as soil and water conservation, which are the result of long-term interactions among climate, landscape, soil, and human activities. It plays an important role in surface energy exchange, the hydrological cycle as well as in climate regulation. Climate change and vegetation are inextricably linked climate change is the driving force behind changes in vegetation, and vegetation is a comprehensive indicator of response to climate change. Vegetation quality is a key indicator of natural ecological conditions reflecting the physical properties of the land surface and the impact of human activities on natural systems in an integrated manner, as well as of the quality of the regional ecological environment.

The factors selected while calculating the VQI of the ESA model include the risk of fire, the protection offered by the vegetation against soil erosion, the degree of resistance to drought shown by the vegetation, and the vegetation cover.

Besides this, the net primary productivity (NPP) is also a key variable when characterizing vegetation activity; it is important for assessing the carrying capacity of ecosystems and understanding the carbon cycle of terrestrial ecosystems^54^. Zhang^36^ reported that the QTP alpine ecosystem presents high NPP and fixes more carbon as well, which will help slow down CO_2_ emissions in the region and mitigate the greenhouse effect. The NPP was, therefore, considered as one of the essential metrics when calculating the quality of vegetation. The vegetation distribution on the QTP presents a more significant vertical differentiation with varied altitudes due to the redistribution of light, heat, and water. Altitude is, hence, included in the analysis and calculation of the quality of vegetation as it is an important auxiliary indicator. The vegetation quality indicators were scored with reference to the study by Salvati et al., and the results are shown in Table 6.

**Table 6:** The scores of various indices of vegetation quality on the QTP.

	Classes	Scores		Classes	Scores
**Vegetation quality**					
Vegetation coverage	< 5	2.0	Slope	Sunny slope	2.5
	5–50	4.0		Half sunny slope	5.0
	50–100	6.0		Half shady slope	7.5
	100–158	8.0		Shady slope	10.0
	> 158	10.0			
Richness	< 0.005	2.0	NPP	< 50	2.0
	0.005–0.01	4.0		50–100	4.0
	0.01–0.02	6.0		100–500	6.0
	0.02–0.22	8.0		500–1000	8.0
	> 0.22	10.0		> 1000	10.0
Vegetation resistance to risk	Desert, swamp, others	2.0	Vegetation resistance to risk	Shrub	8.0
	Grassland, grass, meadow, cultivated vegetation	6.0		Woodland (Oniferous and broad leaved mixed forest, broad-leaved forest)	10.0

The VQI of the QTP was calculated using the scoring results of each factor. The equation for the same was as follows:

3 $$ VQI = (Vc*Vr*Vrr*NPP*A)^{\frac{1}{5}} $$

where *Vc* refers to vegetation coverage, *Vr* to richness of vegetation, *Vrr* denotes ability of vegetation to resist ecological risk, *NPP* signifies the net primary productivity of vegetation, and *A* means the aspect.

According to the calculation, nearly 52.86% of the area on the QTP is LeVQ (lower vegetation quality), with only 4.04% being HeVQ (higher vegetation quality) (Table 7). The vegetation quality shows a gradual trend of growth from the northwest to the southeast. The area of HeVQ is mainly located in the Hengduan mountains and the southern valley of Tibet, where the vegetation is cold-temperate coniferous forest and evergreen broad-leaved forest in the plateau mountains, and wet monsoon rainforest in the eastern part of the tropics. The area of HVQ (high vegetation quality) accounts for 11.34% of the total area and is mainly located in the Hehuang valley, the southeastern part of the Qinghai Plateau, and the Hengduan mountains. The vegetation in these areas is dominated by meadows, herbaceous swamps, scrub, and emergent dwarf forests. The LeVQ region is located between the northwestern and the central parts of the QTP, including the northern Tibetan and Ali plateau, and the north-central area from Aljinshan to the Qilian mountains to the Qinghai Plateau. The vegetation in these areas is dominated by grasslands and sparsely forested shrub steppe (Fig. 1a–d).

**Table 7:** The vegetation quality and its proportion on the QTP (percent).

Vegetation quality		Percentage (percent)
Lower vegetation quality (LeVQ)	< 3.53	52.86
Low quality (LVQ)	3.53–5.02	22.88
General vegetation quality (GVQ)	5.02–6.55	8.89
High vegetation Quality (HVQ)	6.55–7.80	11.34
Higher vegetation Quality (HeVQ)	> 7.80	4.04

It is worth mentioning that the reason behind the poor quality of the vegetation in southeastern QTP is due to the high distribution of crops in these areas. This can be attributed to crops being poorer both in terms of hardiness as well as protecting the surface from erosion, thereby leading to the distribution of large areas of low-quality vegetation.

#### Ecological risk assessment model

Using the three indices mentioned above, an ecological risk assessment model based on the natural factors on QTP was constructed as follows:

4 $$ EAI = \alpha \times CQI_{ij} + \gamma \times VQI_{ij} $$

The random forest method used here is commonly used to identify the importance of indicators. After many experiments, the best model results were obtained in the random forest model when the value of parameter mtry was set to 2 and ntree to 1000, respectively. Therefore, the weighted coefficients of each index were finally determined to be 0.6, 0.2, and 0.2, respectively. Additionally, the goodness of these coefficients reached 92.02% based on the conditions mentioned above.

## Results

### Ecological risk assessment in the base year of the QTP

The ecological risk of the QTP is analyzed according to the EAI model. The parameters for dividing the same were formulated to measure the ecological risk (Table 8).

**Table 8:** Classification of ecological risks in the QTP and their proportions (percent).

Ecologic risk		Proportions (%)
Potential ecologic risk area (PERA)	> 6.18	7.81
Lower ecologic risk area (LeERA)	4.86–6.18	17.71
Low ecologic risk area (LERA)	3.66–4.86	25.37
High ecologic risk area (HERA)	2.66–3.66	32.47
Higher ecologic risk area (HeERA)	< 2.66	16.65

**Figure 2:**
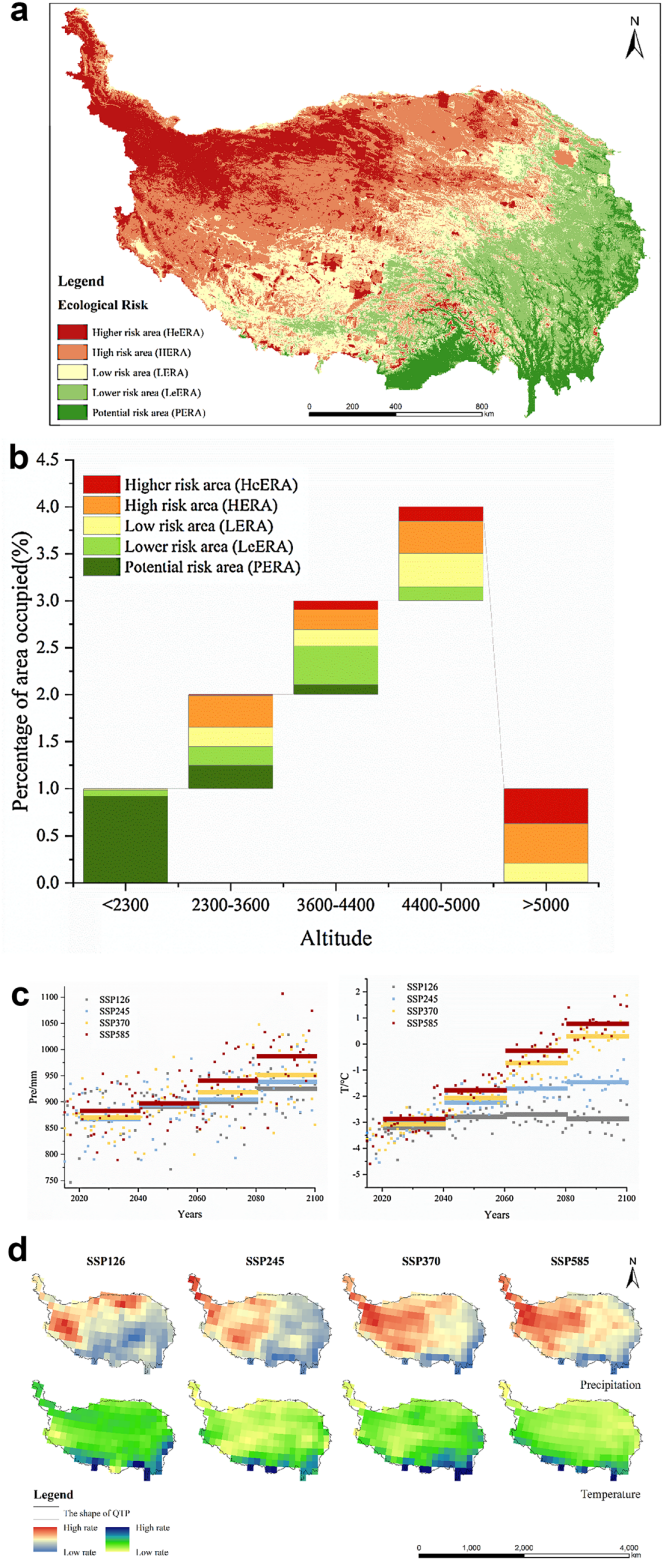

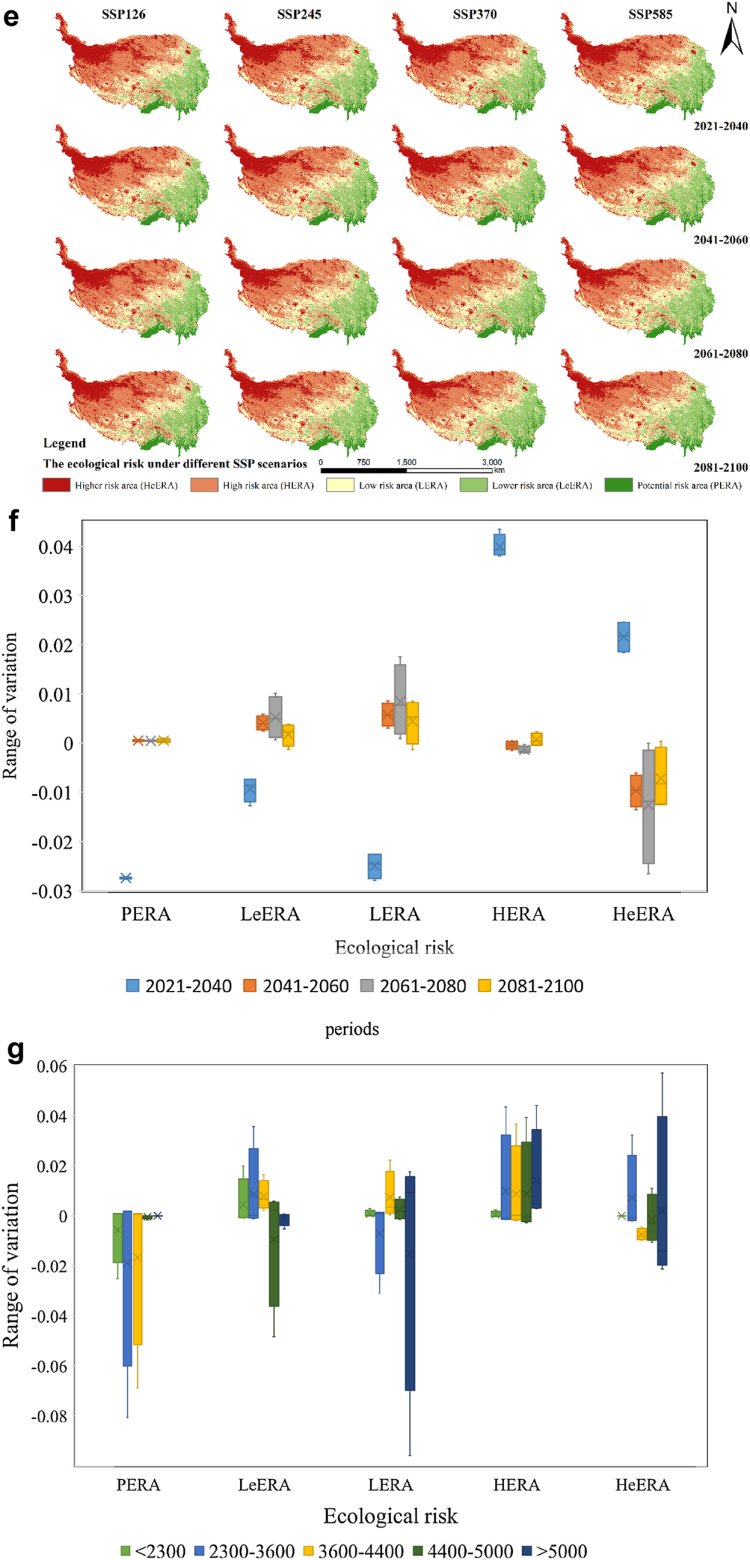
(**a**) The ecological Risk of the QTP (base year). The map was generated by ArcGIS sofware (Version 10.5 for Desktop). (**b**) The proportion of the ecological risk of the QTP at different altitudes (base year). (**c**) Precipitation (mm) (**a**) and average temperature (°C) (**b**) of the QTP under different SSP scenarios from 2021 to 2100. (**d**) The ecological risk of the QTP under different SSP scenarios from 2021 to 2100. The map was generated by ArcGIS sofware (Version 10.5 for Desktop). (**e**) The rate of change of each type of ecological risk from 2021 to 2100. The map was generated by ArcGIS sofware (Version 10.5 for Desktop). (**f**) The rate of change of each type of ecological risk from 2021 to 2100 in different time periods. (**g**) The rate of change of each type of ecological risk from 2021 to 2100 in different elevations.

The evaluation results of the ecological risk in the base year reveal that it is decreasing from northwest to southeast (Figs. 1a, 2a). HeERA (higher ecologic risk area) covers 16.65% of the total area and is mainly located in area near the Kunlun Mountains. The HERA region (high ecologic risk area) on the other hand, covers 32.47% of the total area and is mainly located in the Hehuang valley, the Hengduan mountains, the Songpan Plateau, the southern section of the Qilian Mountains, and the southern region of the Qinghai Plateau. The LERA (low ecologic risk area) region, with an area of 25.24%, is mainly located in the central to southern part of the Qinghai Plateau, the southern part of the Dangla or Tanggula mountain, south of the Northern Tibetan Plateau but north of the Gangdis Mountains, and the Southern Tibetan valley. The area of PERA (potential ecologic risk area) accounts for 8.01% and is mainly located in the Hehuang valley, the southern part of the Songpan Plateau, the Hengduan mountains valley region, and the southern Valley of Tibet (Table 8).

The QTP has a towering terrain and high elevation. According to statistics, the area of the QTP with an altitude of less than 2300 m accounts for 1.97% of the total area, an altitude between 2300 and 3600 m accounts for 16.11% , an altitude between 3600 and 4400 m accounts for 21.52%, an altitude of 4400–5000 m accounts for 37.75%; and an altitude more than 5000 m accounts for 22.65% (Fig. 2b).

Ecological risks show a clear vertical distribution of characteristics of the QTP, and their types differ greatly at different elevations. In the area with an elevation less than 2300 m, the lower the risk level, the larger the area. There is no HeERA area, and HERA only accounts for 0.07% of the total area. LERA accounts for 1.72%, while LeERA reaches 4.17%. The remaining area is all PERA, and the percentage of this area with respect to the total area is 93.03%. On the contrary, there is no presence of PERA in the area located 5000 m above sea level. However, the share of LeERA area decreased to 0.87%, while that of LERA increased to 20.36%; the share of HERA area increased to 41.82%, and that of HeERA increased to 36.95%.

The main types of ecological risks differ at different altitudes. PEAR was the dominant risk type in areas below 2300 m in altitude. For areas with an altitude between 2300 and 3600 m, HERA was the dominant risk type. For areas between 3600 and 4400 m in altitude, LERA dominates instead. Lastly, when the altitude rises above 4400 m, HeERA is the dominant risk type.

However, not all types of risks show a linear increasing trend with increasing altitude. On the whole, with the increase of altitude, the area of PERA exhibits a gradually decreasing trend, the area of HERA and HeERA portray a gradually increasing trend. Both LERA and LeERA show an ascending trend followed by a descending one, with the turning point located between 2300 and 4400 m above sea level.

### Changes in the main climate factors of the QTP in the future

Global temperatures have shown a significant increase in recent years, while severe weather and climate events have exhibited changes in frequency, severity, spatial scale, periodic, as well as time of occurrence^55^. Climate change, which is characterized by rising temperatures, can have dramatic impacts on terrestrial ecosystems^56^.

In order to explore the impact of climate change on ecological risks faced by the QTP, we first need to clarify what this future climate change will look like. Therefore, we calculated and analyzed the BCC-CSM2-MR, CNRM-CM6-1, CNRM-ESM2-1, CanESM5, GFDL-ESM4, IPSL-CM6A-LR, MIROC-ES2 L, MIROC6, and MRI-ESM2-0 modes based on the CMIP6 as well as temperature and precipitation characteristics of the QTP from 2021 to 2100.

According to the analysis, precipitation of the QTP from 2021 to 2100 shows an increase in four scenarios—SSP126, SSP245, SSP370, and SSP585. The rate of increase in precipitation under the SSP126 scenario is about 7.2 mm per decade, 10.5 mm per decade for the SSP245 scenario, about 12.3 mm per decade for the SSP370 scenario, and almost 16.6 mm per decade for the SSP585 scenario making it the highest (Fig. 2c(a)).

The QTP has the same trend of temperature and precipitation, showing the same increasing trend from 2021 to 2100. However, not all four scenarios showed the same consistent trend of elevation. There was an increasing trend in the SSP245, SSP370, and SSP585 scenarios, whereas a decreasing trend in the SSP126 scenario. The temperature increase of QTP in the SSP126 scenario is about 0.07 °C per decade, about 0.3 °C per decade in the SSP245 scenario is, about 0.56 °C per decade in the SSP370 scenario is, and 0.62 °C per decade in the SSP585 scenario, making it the largest increase (Fig. 2c(b); Table 9).

**Table 9:** The rates of temperature and precipitation at QTP in the four scenarios.

	Temperature		Precipitation	
	Rate (℃/10a)	R^2^	Rate/(mm/10a)	R^2^
SSP126	0.07	0.13	7.2	0.15
SSP245	0.28	0.75	10.5	0.27
SSP370	0.56	0.93	12.3	0.30
SSP585	0.62	0.93	16.6	0.44

Although there is an overall trend of increasing precipitation and temperature in QTP, there exist certain distinct regional characteristics as well. The rate of change in temperature showed a gradual increase from northwest to southeast in all four scenarios, while that of precipitation shows an opposite trend—it increases gradually from the northwest to the southeast (Fig. 2d). Ecological risk changes in the QTP, therefore, become more difficult to predict in the present context of an overall increasing, but regionally inconsistent, trend exhibited by climate change.

### Future ecological risk assessment of the QTP

Dynamic indicators of precipitation, temperature, soil moisture, NPP, and other static indicators in the CMIP6 are used to calculate the SQI, CQI, and VQI under the SSP126, SSP245, SSP370 and SSP585 scenarios in the future i.e., from 2021 to 2100. The EAI model is then used to calculate the ecological risk of the QTP from 2021 to 2100.

In general (Fig. 2e), the area of PERA, LeERA, and LERA in QTP decreases significantly and exhibits a clear trend of increasing area in the other two risk categories in the near future, i.e., 2021–2040. Between 2041 and 2060, the area of HERA and HeERA of QTP shows a decreasing trend. The other three types of risks all show an increasing trend when compared to the previous period. The trend of ecological risk change in QTP between 2061 and 2080 is more consistent with the previous period, i.e., 2041 to 2060. However, changes in the SSP126 and SSP245 scenarios are smaller than in the previous period, while the changes in the SSP370 and SSP585 scenarios are larger. Between 2081 and 2100, inconsistent trends emerge. On the other hand, the trends in the SSP245, SSP370, and SSP585 scenarios are more consistent, all showing a decreasing form of HERA and HeERA with the remaining types showing an increase in the area at risk. However, in the SSP126 scenario, both LeERA and LERA show a decreasing trend in area, while the remaining three types exhibit the opposite. Overall, the ecological risk of QTP shows an increasing trend between 2021 and 2040 and then decreases after 2040 (Fig. 2f).

The changes in various types of ecological risks also have different characteristics on different elevation gradients. They are again divided into four time periods in order to consider their change characteristics (Fig. 2g).

From 2021 to 2040, the area of PERA decreases significantly where the altitude is less than 2300 m, decreasing by 2.52% from the base year. The area of LeERA, meanwhile, shows the opposite trend as it increased by 1.99% from the base year. In areas with altitudes between 2300 and 3600 m, PERA and LERA also showed a clear trend of decrease by 8.07% and 3.09%, respectively. In areas with an altitude ranging from 3600 to 4400 m, PERA and HeERA showed a decreasing trend with decreases of 6.88% and 0.67%, respectively. The area of the remaining types of ecological risks, such as HERA, increased significantly. At altitudes of above 4400 m, HERA and HeERA show a clear trend of increase, ranging from 4 to 6%. Meanwhile, the three remaining areas of ecological risk showed a decreasing trend.

These two time periods—2041–2060 and 2061–2080—exhibit a similar trend of change. In areas with an altitude of 4400 m, the changes were not very significant and the rate of change was within 1% despite the area of several types of ecological risks undergoing change. The ecological risk of both HERA and HeERA types decreased more obviously in areas with altitudes above 4400 m. Of these, HeERA showed a larger reduction in area, i.e., between 1 and 2%. Between 2081 and 2100, all areas with altitudes less than 2300 m exhibit a decreasing trend, except for PERA, which slightly increases. The area of both HERA and HeERA types of ecological risk shows a decreasing trend in areas with altitudes between 2300 and 4400. At altitudes above 4400 m, the area of HeERA showed a decreasing trend while those of the remaining types showed a somewhat increasing trend.

Therefore, under different scenarios of SSP, the PERA of QTP shows a decreasing and then increasing trend with an elevation between 2021 and 2040 m. LeERA and HeERA exhibit an increasing, decreasing, and then increasing again trend, and LERA’s trend is opposite to that of LeERA. HERA shows an increasing trend with elevation. From 2041 to 2080, PERA and LeERA show an increasing and then decreasing trend with increasing altitude, while LERA shows an increasing trend with increasing altitude. HeERA shows the opposite trend, while HERA shows an increasing, then decreasing, and then increasing trend with increasing altitude. From 2081 to 2100, the PERA of QTP showed an increasing and then decreasing trend with elevation, while LeERA showed an increasing and then decreasing trend, which then increases again. LERA and HeERA showed the opposite trend.

## Discussion

Assuming that the soil properties will remain unchanged, this paper creates an ecological risk assessment model (EAI) based on climate change. It then uses it to evaluate the ecological risk in the QTP of the present year as well as the next hundred years based on the climate simulation data from various SSP scenarios generated by the IPCC sixth assessment model.

The results of the ecological risk assessment of QTP in the base year show that it is dominated by HERA, which accounts for about 32.47% of the total area, followed by LERA, which accounts for about 25.24% of the area. The ecological risk of QTP has a decreasing trend from northwest to southeast and has a significant vertical distribution feature. As the elevation increases, the area of PERA gradually decreases, the area of HERA and HeERA gradually increases, and the area of LERA and LeERA shows an increasing and then decreasing trend.

The precipitation and temperature in the QTP revealed an overall increasing trend, but with distinct regional characteristics. The rate of change of temperature showed a gradual increase from the northwest to the southeast in all four scenarios. The rate of change of precipitation shows an opposing trend to that of temperature, which gradually increases from the northwest to the southeast. In other words, the temperature increases rapidly in the northwestern part of the QTP, but precipitation increases slowly. Similarly, while the temperature increases slowly in the southeastern part of the QTP, precipitation increases rapidly. With these obvious regional trends, the ecological risk of the QTP becomes difficult to predict.

Therefore, in this paper, the ecological risk of the QTP was simulated and predicted for the period from 2021 to 2100, assuming that no change would occur in the soil structure. The results show that the ecological risk of the QTP shows an increasing trend between 2021 and 2040, and then decreases after 2040. It also shows that the changes of various ecological risks were different at different elevation gradients. Under the different scenarios of SSP, the PERA of QTP showed a decreasing and then increasing trend between 2021 and 2040. LeERA and HeERA showed an increasing and then decreasing trend, which then increased again. LERA showed a trend opposite to that of LeERA, while HERA showed a trend that increased with altitude. From 2041 to 2080, PERA and LeERA show a trend that increases and then decreases with elevation, LERA displayed a trend that increases with elevation, and HeERA revealed the opposite trend—an increasing, then decreasing trend that increases again with elevation. From 2081 to 2100, PERA of the QTP showed a trend that increased and then decreased with elevation, while LeERA showed a trend that increases, then decreases, and then increased again. LERA and HeERA, therefore, showed opposing trends.

That the overall ecological risk of QTP tends to increase between 2021 and 2100, but with different characteristics at different altitudes, is an interesting conclusion. The overall ecological risk tends to increase in areas less than 3600 m above sea level. In areas ranging between 3600 and 5000 m above sea level, PREA and HeERA showed a decreasing trend, while the area of the rest of the risk categories exhibited an increasing trend. In the area with an altitude over 5000 m, HeERA and HERA showed more obvious increases, while the area of the remains risk area showed a decreasing trend. Overall, it appears that the ecological risk of the QTP will change in the next 80 years and that the specific trend of the ecological risk of Qing QTP can be further clarified by studying the interconversion between each risk in future studies.

## Code and data availability

All codes to process the data (R code) and the results themselves are available upon request from the corresponding author.
